# Use of the Pfizer Pentavalent Meningococcal Vaccine Among Persons Aged ≥10 Years: Recommendations of the Advisory Committee on Immunization Practices ― United States, 2023

**DOI:** 10.15585/mmwr.mm7315a4

**Published:** 2024-04-18

**Authors:** Jennifer P. Collins, Samuel J. Crowe, Ismael R. Ortega-Sanchez, Lynn Bahta, Doug Campos-Outcalt, Jamie Loehr, Rebecca L. Morgan, Katherine A. Poehling, Lucy A. McNamara

**Affiliations:** ^1^Division of Bacterial Diseases, National Center for Immunization and Respiratory Diseases, CDC; ^2^Coronavirus and Other Respiratory Viruses Division, National Center for Immunization and Respiratory Diseases, CDC; ^3^Minnesota Department of Health; ^4^College of Medicine and Public Health, University of Arizona, Phoenix, Arizona; ^5^Cayuga Family Medicine, Ithaca, New York; ^6^Department of Health Research Methods, Evidence, and Impact, McMaster University, Hamilton, Ontario, Canada; ^7^Wake Forest University School of Medicine, Winston-Salem, North Carolina.

**Summary**

**What is already known about this topic?**

Meningococcal disease is a life-threatening invasive infection caused by *Neisseria meningitidis*. The pentavalent meningococcal vaccine (MenACWY-TT/MenB-FHbp [Penbraya, Pfizer Inc.]) protects against *N. meningitidis* serogroups A, B, C, W, and Y and is licensed for use among persons aged 10–25 years.

**What is added by this report?**

On October 25, 2023, the Advisory Committee on Immunization Practices recommended that MenACWY-TT/MenB-FHbp may be administered to persons aged ≥10 years when both a quadrivalent meningococcal conjugate vaccine (MenACWY) and meningococcal B vaccine (MenB) are indicated at the same visit.

**What are the implications for public health practice?**

MenACWY-TT/MenB-FHbp is the first pentavalent meningococcal vaccine approved for protection against serogroups A, B, C, W, and Y. Different manufacturers’ MenB vaccines are not interchangeable; when MenACWY-TT/MenB-FHbp is administered, subsequent doses of MenB should be from the same manufacturer (Pfizer Inc.).

## Abstract

Meningococcal disease is a life-threatening invasive infection caused by *Neisseria* *meningitidis*. Two quadrivalent (serogroups A, C, W, and Y) meningococcal conjugate vaccines (MenACWY) (MenACWY-CRM [Menveo, GSK] and MenACWY-TT [MenQuadfi, Sanofi Pasteur]) and two serogroup B meningococcal vaccines (MenB) (MenB-4C [Bexsero, GSK] and MenB-FHbp [Trumenba, Pfizer Inc.]), are licensed and available in the United States and have been recommended by CDC’s Advisory Committee on Immunization Practices (ACIP). On October 20, 2023, the Food and Drug Administration approved the use of a pentavalent meningococcal vaccine (MenACWY-TT/MenB-FHbp [Penbraya, Pfizer Inc.]) for prevention of invasive disease caused by *N. meningitidis* serogroups A, B, C, W, and Y among persons aged 10–25 years. On October 25, 2023, ACIP recommended that MenACWY-TT/MenB-FHbp may be used when both MenACWY and MenB are indicated at the same visit for the following groups: 1) healthy persons aged 16–23 years (routine schedule) when shared clinical decision-making favors administration of MenB vaccine, and 2) persons aged ≥10 years who are at increased risk for meningococcal disease (e.g., because of persistent complement deficiencies, complement inhibitor use, or functional or anatomic asplenia). Different manufacturers’ serogroup B–containing vaccines are not interchangeable; therefore, when MenACWY-TT/MenB-FHbp is used, subsequent doses of MenB should be from the same manufacturer (Pfizer Inc.). This report summarizes evidence considered for these recommendations and provides clinical guidance for the use of MenACWY-TT/MenB-FHbp.

## Introduction

Meningococcal disease is a life-threatening invasive infection caused by *Neisseria meningitidis*. CDC’s Advisory Committee on Immunization Practices (ACIP) recommends routine administration of a single dose of quadrivalent (serogroups A, C, W, and Y) meningococcal conjugate vaccine (MenACWY) to persons at age 11 or 12 years, with a booster dose at age 16 years. ACIP recommends a 2-dose serogroup B meningococcal vaccine (MenB) series for persons aged 16–23 years, based on shared clinical decision-making, to provide short-term protection against meningococcal disease caused by most serogroup B strains (*1*). ACIP also recommends routine vaccination with MenACWY (for persons aged ≥2 months) and MenB (for persons aged ≥10 years) who are at increased risk for meningococcal disease caused by the serogroups covered by each vaccine (Box) (*1*).

BOX Existing Meningococcal Vaccination Recommendations* — Advisory Committee on Immunization Practices, United States, 2024

**ACIP recommends MenACWY vaccination for the following groups:**

- Routine vaccination for persons aged 11 or 12 years, with a booster dose at age 16 years
- Routine and booster vaccination of persons aged ≥2 months at increased risk for meningococcal disease (dosing schedule varies by age and indication, and interval for booster doses varies by age at time of previous vaccination):
  - Persons with certain medical conditions including anatomic or functional asplenia, complement component deficiencies (e.g., C3, C5–C9, properdin, factor H, or factor D), complement inhibitor (e.g., eculizumab [Soliris] or ravulizumab [Ultomiris]) use, or HIV infection
  - Microbiologists with routine exposure to *Neisseria meningitidis* isolates
  - Persons at increased risk during an outbreak (e.g., in community or organizational settings, and among men who have sex with men)
  - Persons who travel to or live in countries where meningococcal disease is hyperendemic or epidemic
  - Unvaccinated or undervaccinated first-year college students living in residence halls
  - Military recruits

**ACIP recommends MenB vaccination for the following groups:**

- Routine and booster vaccination of persons aged ≥10 years at increased risk for meningococcal disease (dosing schedule varies by vaccine brand; boosters should be administered at 1 year after primary series completion, then every 2–3 years thereafter for those who remain at increased risk):
  - Persons with certain medical conditions, such as anatomic or functional asplenia, complement component deficiencies, or complement inhibitor use
  - Microbiologists with routine exposure to *N. meningitidis* isolates
  - Persons at increased risk during an outbreak (e.g., in community or organizational settings, and among men who have sex with men)
- Vaccination of adolescents and young adults aged 16–23 years with a 2-dose MenB series on the basis of shared clinical decision-making. The preferred age for MenB vaccination is 16–18 years

**Abbreviations:** ACIP = Advisory Committee on Immunization Practices; MenACWY = quadrivalent (serogroups A, C, W, and Y) meningococcal vaccine; MenB = serogroup B meningococcal vaccine.

* https://pubmed.ncbi.nlm.nih.gov/33417592/

In October 2023, a pentavalent meningococcal vaccine (MenACWY-TT/MenB-FHbp [Penbraya, Pfizer Inc.]) was licensed for use in persons aged 10–25 years (*2*). MenACWY-TT/MenB-FHbp contains the same components as those in two existing meningococcal vaccines: 1) *N. meningitidis* polysaccharide groups A, C, W, and Y conjugated to tetanus toxoid carrier protein (MenACWY-TT* [Nimenrix, Pfizer Inc.], a non–U.S.-licensed vaccine), and 2) two recombinant lipidated factor H–binding protein (FHbp) variants from *N. meningitidis* serogroup B (MenB-FHbp [Trumenba, Pfizer Inc.]). This report summarizes evidence considered for these recommendations and provides clinical guidance for the use of MenACWY-TT/MenB-FHbp.

## Methods

During June 2022–October 2023, the ACIP Meningococcal Vaccines Work Group held monthly conference calls to review meningococcal disease epidemiology and evidence regarding use of MenACWY-TT/MenB-FHbp in persons currently recommended to receive MenACWY and MenB (policy question 1), MenACWY only (policy question 2), or MenB only (policy question 3). To guide deliberations, ACIP used the Evidence to Recommendations framework and considered the importance of meningococcal disease as a public health problem, benefits, and harms of MenACWY-TT/MenB-FHbp, values of the target population, acceptability, resource use, equity, and feasibility.^†^ ACIP evaluated the available evidence on the following prespecified benefits and harms (each with ranked importance), using the Grading of Recommendations, Assessment, Development and Evaluation (GRADE) approach (*3*): disease caused by serogroups A, B, C, W, and Y (critical); short-term immunity (critical); persistent immunity (important); serious adverse events (critical); nonserious adverse events (important); and interference with other recommended vaccines administered concurrently (important).^§^

## Summary of Evidence for Use of MenACWY-TT/MenB-FHbp in Persons Aged ≥10 Years

### Safety and Immunogenicity

The body of evidence comprised data from three randomized, quadruple-blinded multisite^¶^ clinical trials that assessed immunogenicity and safety** among healthy participants aged 10–25 years. Participants were randomized to 1) the pentavalent group (2 doses of MenACWY-TT/MenB-FHbp, administered 6 or 12 months apart^††^) or 2) the control group (MenACWY-CRM [Menveo, GSK, 1 dose] + MenB-FHbp [2 doses administered 6 months apart]) (*4*). The trials included ACWY-naive and ACWY-primed participants; all study participants were MenB-naive. The GRADE assessment focused on the 6-month pentavalent dosing interval for immunity outcomes; data on both 6- and 12-month pentavalent dosing intervals were assessed for safety outcomes.

### Short-Term Immunity

 Among both MenACWY-naive and MenACWY-primed participants, seroresponse^§§^ for serogroups A, C, W, and Y 1 month after the first trial dose of ACWY-containing vaccine was achieved as often or more often in the pentavalent group than in the control group. On the basis of a composite measure, seroresponse for serogroup B 1 month after the second dose of serogroup B–containing vaccine was achieved more often in the pentavalent group than in the control group. The overall level of certainty for the critical outcome short-term immunity for all serogroups was moderate for healthy persons and low for persons at increased risk because of underlying medical conditions.

### Persistent Immunity

Among ACWY-naive and ACWY-primed participants, seroprotection^¶¶^ for meningococcal serogroups A, C, W, and Y occurred as often or more often in the pentavalent group (48 months after receipt of 2 doses MenACWY-TT/MenB-FHbp) compared with the control group (54 months after 1 dose MenACWY-CRM). Little or no difference was observed in the frequency of serogroup B strain–specific seroprotection*** 48 months after receipt of 2 doses of pentavalent vaccine when compared with those seen 48 months after receipt of 2 doses of MenB-FHbp + 1 dose MenACWY-CRM. The overall level of certainty for this important outcome was low for serogroups A, C, W, and Y for healthy persons, moderate for serogroup B for healthy persons, and low for all serogroups for those at increased risk because of underlying medical conditions.

### Adverse Events

The proportion of participants who experienced serious adverse events^†††^ was similar in the pentavalent group (0.6%) and the control group (0.5%; p = 0.7). No serious adverse events were deemed related to the vaccine by the study investigators. The pentavalent group had significantly fewer nonserious adverse events^§§§^ (24.6%) than did the control group (32.5%; p<0.001). The most common solicited adverse events within 7 days after receipt of either trial dose of MenACWY-TT/MenB-FHbp were injection site pain (84.4%–89.3%; mostly mild or moderate), fatigue (47.6%–52.1%; mostly mild or moderate), and headache (39.8%–46.8%; mostly mild or moderate) (*5*). For both serious and nonserious adverse events, the level of certainty was low for healthy persons and very low for those at increased risk because of underlying medical conditions.

### Coadministration with Other Vaccines

No data exist on coadministration of MenACWY-TT/MenB-FHbp with other vaccines. Review of the interactions sections of the package inserts for the component vaccines Nimenrix (MenACWY-TT) and Trumenba (MenB-FHbp) did not identify any concerns for coadministration with other vaccines (*6*,*7*).

### Resource Use

Findings from two economic models (CDC model and Pfizer Inc. model) that assessed the health benefits and cost-effectiveness of MenACWY-TT/MenB-FHbp for each policy question within the routine schedule were considered by ACIP (*8*). According to the CDC model, strategies likely to be societally cost-saving would use the pentavalent vaccine to 1) replace a single dose of MenACWY and MenB when both are indicated, or 2) replace MenACWY and MenB when both are indicated, followed by completion of the 2-dose MenB series with a second dose of pentavalent vaccine. The CDC model also illustrated that when immunization against serogroup B meningococcal disease is not indicated, replacing both doses of MenACWY with the pentavalent vaccine would be incrementally less cost-effective. Despite differences in input values and assumptions, similar conclusions were reported by the Pfizer Inc. model.

## Recommendations for Use of MenACWY-TT/MenB-FHbp

ACIP recommended that MenACWY-TT/MenB-FHbp may be used when both MenACWY and MenB are indicated at the same visit for 1) healthy persons aged 16–23 years (routine schedule) when shared clinical decision-making favors administration of MenB vaccine and 2) persons aged ≥10 years who are at increased risk for meningococcal disease (e.g., because of persistent complement deficiencies, complement inhibitor use, or functional or anatomic asplenia) (Table) (Figure). Indications for MenACWY and MenB vaccination have not changed since they were previously published (*1*).

**TABLE:** Recommended timing of meningococcal vaccine doses*^,^^†^ within the routine schedule^§^ based on the outcome of shared clinical decision-making regarding meningococcal B vaccine — United States, 2023

Recipient age group, yrs	Recommendation based on shared clinical decision-making for MenB		
	MenB not favored	MenB favored at age 16 yrs	MenB favored at age >16 yrs
**11–12**	MenACWY dose #1	MenACWY dose #1	MenACWY dose #1
**16**	MenACWY dose #2	MenACWY dose #2 + MenB-4C^¶ ^or MenACWY dose #2 + MenB-FHbp**^ ^or MenACWY-TT/MenB-FHbp followed by MenB-FHbp 6 mos later	MenACWY dose #2
**17–23**	NA	NA	MenB-4C^¶ ^or MenB-FHbp**

**Abbreviations:** MenACWY = quadrivalent (serogroups A, C, W, and Y) meningococcal conjugate vaccine; MenACWY-TT/MenB-FHbp = Penbraya (Pfizer Inc.) pentavalent (serogroups A, B, C, W, and Y) meningococcal vaccine; MenB-FHbp = Trumenba (Pfizer Inc.) serogroup B meningococcal vaccine; MenB-4C = Bexsero (GSK) serogroup B meningococcal vaccine; NA = not applicable.

* Assumes that a person has not previously been vaccinated with MenACWY or MenB.

^†^ MenACWY vaccines are interchangeable; the same vaccine product is recommended, but not required, for all doses. Different manufacturers’ MenB vaccines are not interchangeable. https://pubmed.ncbi.nlm.nih.gov/33417592/

^§^ To determine catch-up vaccination recommendations for MenACWY and MenB, clinicians should see previously published recommendations. https://pubmed.ncbi.nlm.nih.gov/33417592/

^¶^ Two-dose series with doses administered ≥1 month apart.

** Two-dose series with doses administered 6 months apart.

**FIGURE:**
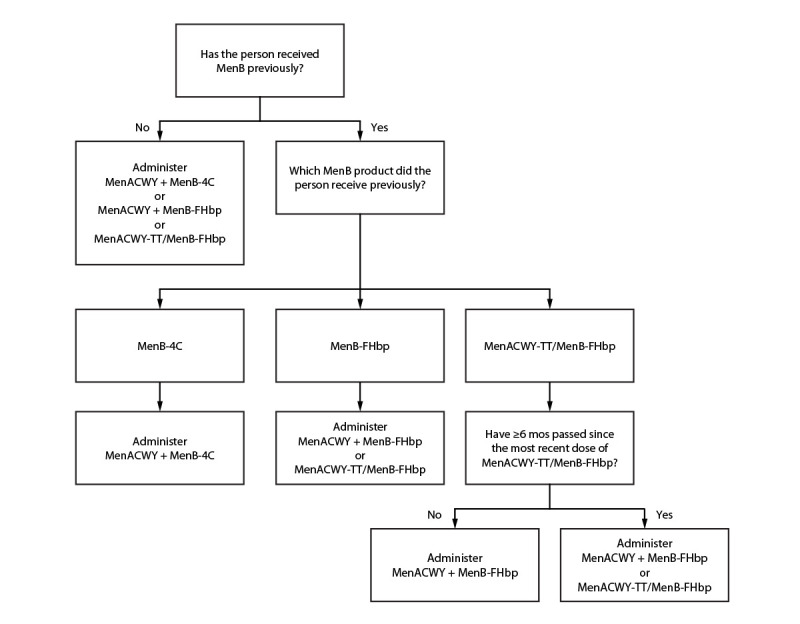
Recommended meningococcal vaccines for persons at increased risk for meningococcal disease due to serogroups A, B, C, W, or Y and who are due for both meningococcal A, C, W, and Y vaccine* and meningococcal B vaccine^†^^,^^§^^,^^¶^^,^** — United States, 2023 **Abbreviations:** MenACWY = quadrivalent (serogroups A, C, W, and Y) meningococcal conjugate vaccine; MenACWY-TT/MenB-FHbp = Penbraya (Pfizer Inc.) pentavalent (serogroups A, B, C, W, and Y) meningococcal vaccine; MenB-FHbp = Trumenba (Pfizer Inc.) serogroup B meningococcal vaccine; MenB-4C = Bexsero (GSK) serogroup B meningococcal vaccine. * MenACWY products are interchangeable; the same vaccine product is recommended, but not required, for all doses. ^†^ Different manufacturers’ MenB vaccines are not interchangeable. ^§^ To determine whether MenACWY and MenB are indicated based on a person’s risk factors and timing of any previous meningococcal vaccines, clinicians should see previously published recommendations. https://pubmed.ncbi.nlm.nih.gov/33417592/ ^¶^ If MenB was received previously but the vaccine manufacturer is not known, the series must be restarted with any licensed product to ensure completion of the series using products from a single manufacturer. For additional guidance, clinicians should see previously published recommendations. https://pubmed.ncbi.nlm.nih.gov/33417592/ ** If MenB-FHbp was received previously, MenACWY-TT/MenB-FHbp may be used provided the person has not received MenACWY-TT/MenB-FHbp previously or ≥6 months have passed since the previous dose of MenACWY-TT/MenB-FHbp.

## Clinical Guidance

### Shared Clinical Decision-Making for MenB

For healthy persons, use of MenACWY-TT/MenB-FHbp should not supersede discussion of whether to administer MenB using shared clinical decision-making (Table). Clinicians should refer to previously published considerations for shared clinical decision-making and timing of MenB administration (*1*).

### Interchangeability of Vaccine Products

MenACWY products are interchangeable; the same vaccine product is recommended, but not required, for all doses (*1*). Different manufacturers’ MenB products are not interchangeable; administration of a B-component vaccine (monovalent or pentavalent) requires that all subsequent B-component vaccine doses, including booster doses, be from the same manufacturer. If one MenB dose was received but the vaccine manufacturer is not known, the series must be restarted with any licensed product to ensure completion of the MenB series using products from a single manufacturer.

If MenACWY-TT/MenB-FHbp is inadvertently administered in lieu of MenACWY or MenB when only one (i.e., MenACWY or MenB) was indicated, the dose can be considered valid if it would otherwise have been a valid dose of MenACWY or MenB (i.e., on the basis of indication, patient age, and dosing interval).

### Dosing Intervals

The licensed dosing interval for MenACWY-TT/MenB-FHbp is 6 months. Data are not available regarding safety or immunogenicity of MenACWY-TT/MenB-FHbp with dosing intervals exceeding 12 months. Healthy adolescents and young adults aged 16–23 years who receive 1 dose of MenACWY-TT/MenB-FHbp on the basis of shared clinical decision-making should complete the MenB series with a dose of MenB-FHbp 6 months after the pentavalent vaccine dose was administered (Table).

Persons at increased risk for meningococcal disease who receive a dose of MenACWY-TT/MenB-FHbp and are recommended to receive additional doses of MenACWY and MenB <6 months after a dose of pentavalent meningococcal vaccine should receive separate MenACWY and MenB-FHbp vaccines rather than MenACWY-TT/MenB-FHbp (Figure). MenACWY-TT/MenB-FHbp may be used for booster doses in persons who remain at increased risk if a booster dose of both MenACWY and MenB are indicated at the same visit. MenACWY-TT/MenB-FHbp doses deviating from the licensed 6-month interval can be considered valid for MenACWY or MenB if the timing would otherwise have been valid for that component.

### Contraindications and Precautions

**Severe allergy.** MenACWY-TT/MenB-FHbp is contraindicated for persons with a history of severe allergic reaction, such as anaphylaxis, to any component of the vaccine or to a tetanus toxoid–containing vaccine.

**Pregnancy and breastfeeding.** No data exist on use of MenACWY-TT/MenB-FHbp during pregnancy or while breastfeeding. Because limited data are available for MenB vaccination during pregnancy, vaccination with MenB should be deferred unless the pregnant person is at increased risk for acquiring meningococcal disease, and, after consultation with their health care provider, the benefits of vaccination are considered to outweigh the potential risks. When MenACWY is indicated, persons who are pregnant or breastfeeding should receive MenACWY-CRM or MenACWY-TT (MenQuadfi, Sanofi Pasteur).

### Reporting of Vaccine Adverse Events

Adverse events that occur in a patient after meningococcal vaccination should be reported to the Vaccine Adverse Event Reporting System (VAERS), even if it is uncertain whether the vaccine caused the event. Instructions for reporting to VAERS are available online at https://vaers.hhs.gov/reportevent.html or by telephone (800-822-7967).

## Footnotes

* Nimenrix and MenQuadfi (both abbreviated MenACWY-TT) are different vaccines containing different amounts of tetanus toxoid conjugate.

†
https://www.cdc.gov/vaccines/acip/recs/grade/mening-MenACWY-TT-MenB-FHbp-etr.html

§
https://www.cdc.gov/vaccines/acip/recs/grade/mening-MenACWY-TT-MenB-FHbp.html

¶ The clinical trials were conducted in Czechia, Denmark, Finland, Hungary, Poland, and the United States.

** No data were available to assess the outcomes of disease caused by serogroups A, B, C, W, and Y, or interference with vaccines administered concurrently.

†† Two trials evaluated 2 doses of MenACWY-TT/MenB-FHbp administered 6 months apart. One trial evaluated 2 doses of MenACWY-TT/MenB-FHbp administered 12 or 36 months apart; results were not available for the 36-month interval before the October 2023 ACIP vote.

§§ Seroresponse was based on serum bactericidal antibody assays using human complement (hSBA). For participants with a baseline hSBA titer <1:4, seroresponse was defined as a titer ≥1:16. For those with a baseline hSBA titer ≥1:4 and <1:8 (<1:16 for FHbp type A22), seroresponse was defined as a titer ≥1:32 (≥1:64 for A22). For those with a baseline hSBA titer ≥1:8 (≥1:16 for A22), seroresponse was defined as a titer ≥4 times the baseline titer.

¶¶ Seroprotection was based on serum bactericidal antibody assays using hSBA and defined as achieving an hSBA titer ≥1:8 for serogroups A, C, W, and Y.

*** Seroprotection was based on serum bactericidal antibody assays using hSBA and defined as an hSBA titer ≥1:8 for serogroup B (≥1:16 for strain A22).

†††
https://www.fda.gov/safety/reporting-serious-problems-fda/what-serious-adverse-event

§§§ For GRADE, nonserious adverse events included all adverse events during the vaccination phase except for serious adverse events. As defined by Pfizer Inc., all adverse events included serious adverse events, nonserious adverse events, medically attended events (nonserious adverse events that resulted in evaluation at a medical facility), and newly diagnosed chronic medical conditions (a disease or medical condition, not previously identified, that is expected to be persistent or is otherwise long-lasting in its effects). The vaccination phase was defined as the time from receipt of the first study vaccination through 1 month receipt after the second study vaccination.
